# Continuous intraoperative neuromonitoring (cIONM) in head and neck surgery—a review

**DOI:** 10.1007/s00106-020-00824-1

**Published:** 2020-03-26

**Authors:** P. Stankovic, J. Wittlinger, R. Georgiew, N. Dominas, S. Hoch, T. Wilhelm

**Affiliations:** 1 Department of Otolaryngology, Head/Neck & Facial Plastic Surgery, Sana Kliniken Leipziger Land, Rudolf-Virchow-Straße 2, 04552 Borna, Germany; 2 Department of Otolaryngology, Head and Neck Surgery, Martin Luther-University Halle-Wittenberg, Halle (Saale), Germany; 3 Department of Otolaryngology, Philipps-University Marburg, Marburg, Germany; 4 Medical Faculty, Philipps-University Marburg, Marburg, Germany

**Keywords:** Cerebellopontine angle, Thyroidectomy, Intraoperative neurophysiological monitoring, Facial nerve, Vagal nerve

## Abstract

Although the history of intraoperative neuromonitoring (IONM) dates back to the 19th century, the method did not evolve further than the mere differentiation of nerves until recently. Only the development of continuous IONM (cIONM) has allowed for non-stop analysis of excitation amplitude and latency during surgical procedures, which is nowadays integrated into the software of almost all commercially available neuromonitoring devices. The objective of cIONM is real-time monitoring of nerve status in order to recognize and prevent impending nerve injury and predict postoperative nerve function. Despite some drawbacks such as false-positive/negative alarms, technical artefacts, and rare adverse effects, cIONM remains a good instrument which is still under development. Active (acIONM) and passive (pcIONM) methods of cIONM are described in literature. The main fields of cIONM implementation are currently thyroid surgery (in which the vagal nerve is continuously stimulated) and surgery to the cerebellopontine angle (in which the facial nerve is either continuously stimulated or the discharge signal of the nerve is analyzed via pcIONM). In the latter surgery, continuous monitoring of the cochlear nerve is also established.

Intraoperative neuromonitoring (IONM) aims to anatomically and functionally preserve neural structures at risk, thus avoiding postoperative short- and long-term paresis. Modern IONM enables not only nerve identification but also the avoidance of harmful preparation and gives a prognostic quality to intraoperative information. Applications are common in otolaryngology, neurosurgery, and general surgery.

## Background

The history of IONM began in the 1898 when Dr. Fedor Krause from Berlin, Germany, used monopolar faradic stimulation during an acoustic nerve neurectomy [13]. In order to check the function of the facial nerve after performing a cochlear nerve section for uncontrollable tinnitus, Krause stimulated the facial nerve and visually noted “… contractions of the facial region, especially of the orbicularis oculi, as well as the branches supplying the nose and the mouth.” The breakthrough years for IONM came in the 1960s when Flisberg and Lindholm [7] introduced it to thyroid surgery, and Parsons and Hilger, in different studies, devised facial nerve stimulators for use in parotid and ear surgery [10, 20].

In recent decades, the use of IONM has become standard in many institutions where it is used mainly as an identifying tool during careful dissection in the proximity of the nerve. The main surgical fields in which it is employed are surgeries on the thyroid gland where the vagus nerve and recurrent laryngeal nerve are monitored, parotidectomy, and surgery of the posterior cranial fossa where the facial nerve is monitored.

During the course of the operation, the surgeon uses a stimulator probe in order to identify the nerve and differentiate it from other tissue. The probe is placed onto the nerve, thus closing a circuit and producing further visual or acoustic stimuli every time the nerve is touched. Such application can be defined as intermittent IONM (iIONM).

Modern IONM has prognostic implications for intraoperative information

In otolaryngology, iIONM is widespread in parotid surgery. Some authors have reported lower postoperative facial palsy rates when using iIONM [16, 27, 35]. On the other hand, there are studies that state that iIONM does not lead to less paresis after surgery [4, 9, 40]. In the United States, there is no clear recommendation on whether a head and neck surgeon should use iIONM when performing surgeries on the parotid gland. As a result, 40% of otolaryngologists in United States do not currently routinely use iIONM, but rely rather on their surgical skills and knowledge of anatomical landmarks [17]. A recent meta-analysis comparing parotidectomies with and without iIONM found the incidence of immediate postoperative facial nerve palsy following parotidectomy while using iIONM to be significantly lower when compared with that of no intraoperative monitoring [34].

The aim of cIONM is to provide real-time monitoring of the nerve status during exposure

Recently, the development of IONM has been enhanced by the establishment of so-called continuous intraoperative neuromonitoring (cIONM) in clinical practice. This aims to provide real-time monitoring of the nerve status during the entire course of exposure and, perhaps more importantly, to predict postoperative function. This is done either actively (acIONM) or passively (pcIONM; Table 1). During acIONM, the nerve is stimulated continuously during the whole procedure. This can be facilitated using either an electrode that is placed on the nerve or near the nerve, by transcranial nerve stimulation or, in the case of vestibulocochlear monitoring, using acoustic stimuli at a given frequency (Hz). Active cIONM has been developed in thyroid surgery [5, 8, 12, 14, 21, 25, 30, 32, 33], surgery on the posterior cranial fossa [1, 2, 37, 41], and surgery on vascular anomalies [38] where vagus, facial, and vestibulocochlear nerves are at risk.

**Table 1:** Classification of continuous intraoperative neuromonitoring (cIONM) according to the stimulation site

**Active continuous intraoperative neuromonitoring (acIONM)**
*Facial nerve (N. VII)*
– Direct (surgery of the posterior cranial fossa)
– Percutaneous (surgery of facial malformation)
– Transcranial (electrical stimulation of the corticobulbar tract in the surgery of the posterior cranial fossa)
*Vestibulocochlear nerve (N. VIII)*
– Direct acoustic (surgery of the posterior cranial fossa)
*Vagus nerve (N. X)*
– Direct (thyroid surgery)
**Passive continuous intraoperative neuromonitoring (pcIONM)**
*Facial nerve (N. VII)*
– Free-running, processed discharge electromyogram (EMG)

It is important, at this point, to differentiate between iIONM and cIONM, because many authors refer to iIONM as continuous, thus resulting in some confusion. During iIONM, the patient is indeed continuously “attached” to the monitoring device, but the information that the surgeon receives is in fact not continuous. In this setting, only the identification of the nerve from the surrounding tissue is possible and only when the surgeon actively makes use of the stimulator probe. The nerve amplitudes and latencies are not subject to analysis. This means that for the majority of intraoperative time “what the nerve has to say is not heard.” During cIONM, on the other hand, uninterrupted analysis of amplitude and latency of the nerve is “fed” into a monitoring device, enabling computerized analysis.

## The benefits of cIONM

### acIONM of the vagus nerve in thyroid surgery

**Fig. 1:**
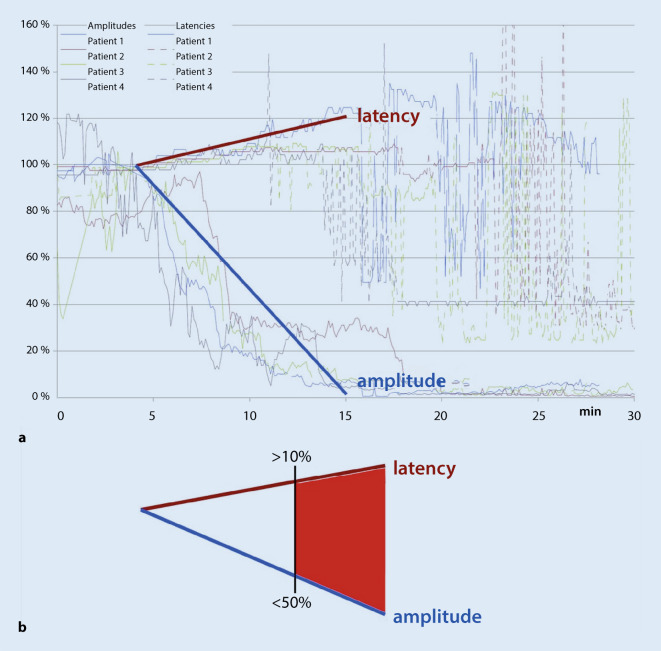
**a** Complete loss of signal during active continuous intraoperative neuromonitoring of the vagus in thyroid surgery resulting in postoperative recurrent laryngeal nerve palsy. **b** Schematic cut-offs for postoperative impaired nerve function. (From [32]. Reprinted with permission © John Wiley and Sons)

Five different modalities of acIONM are described in the literature (Table 1). Active cIONM is facilitated by an electrode placed on the vagus nerve between the common carotid artery and internal jugular vein and is used to monitor the functioning of the recurrent vagus nerve. The nerve has to be dissected from the carotid sheet and have 360° exposure in order to position the electrode. Here, a pattern of impending nerve injury has been identified [32]. It has been noted that decreases of amplitude or latency alone do not have a prognostic factor. However, a combination of a deterioration of amplitude by more than 50% and prolonged latency by more than 10%, namely, multiple combined events (mCE), precedes complete loss of signal (LOS; decline of the amplitude to less than 100 μV) and thus predicts postoperative vocal cord palsy (VCP; Fig. 1).

When the mCE does not evolve to an LOS, the postoperative function of the nerve remains normal. Therefore, when a surgeon notes mCE during surgery and performs an avoidance maneuver, for example, reduced traction, postoperative palsy can be avoided. This is a novel method of avoiding impending nerve injury.

During iIONM, most of the time “what the nerve has to say is not heard”

Bearing in mind that a neurophysiological pattern of nerve injury can be seen during cIONM, a study was published that compared cIONM and iIONM in 1526 consecutive patients undergoing thyroid surgery, split into groups of a similar size [33]. Continuous IONM demonstrated a statistically significant difference with regard to permanent VCP: There were no cases of permanent VCP in the cIONM group whereas four cases of permanent VCP were noted in the iIONM group. This study showed an important benefit of cIONM: In the cIONM patients observed, 77 mCEs were noted, 63 of which (82%) could be actively reversed by the surgeon by stopping the suspected causative maneuver [33].

Furthermore, Schneider et al. demonstrated in their prospective, multicentric study an excellent prognosis for postoperative vocal fold function relative to vagus amplitude recovery of ≥50% after LOS [31]. In bilateral thyroid resection, this information helps decision-making regarding whether the other side should be resected or not when LOS is encountered on the first side. If the LOS recovers to ≥50% of the baseline amplitude, the resection on the contralateral side can be performed safely.

### Direct acIONM in posterior cranial fossa surgery

Direct stimulation of the facial nerve during posterior cranial fossa surgery was described by Amano [1]. The author used a ball-type electrode to directly stimulate the root exit zone of the nerve and secured it with cotton pads. Significant differences according to the House–Brackmann (HB) grade regarding the last maximal amplitude as well as the amplitude preservation ratio (last amplitude at the end of resection compared with the baseline amplitude) were noted between the groups of different facial nerve palsy. Furthermore, it was reported that patients with good postoperative functional results according to the HB grade, along with patients having long-term postoperative improvement in HB grade, showed statistically higher amplitude preservation rates [1]. Therefore, acIONM was demonstrated to be potentially useful in surgery of the posterior cranial fossa with regard to the facial nerve.

### acIONM in vascular malformation surgery

Another extracranial method of facial nerve neuromonitoring is percutaneous stimulation. Ulkatan and colleagues used two monopolar EMG needles during surgery on facial vascular malformations, introducing them percutaneously toward the stylomastoid foramen [38]. The electrodes were used as stimulator probes for preoperative percutaneous mapping of the facial nerve, acIONM, and intraoperative mapping of the facial nerve. In 161 mostly young patients (mean age: 14 ± 14 years), acIONM enabled preoperative nerve mapping, mainly in patients where face edema due to preoperative sclerotherapy masked muscle twitches; needle placement in all surgeries was achieved without complications [38]. A baseline value of the compound muscle action potential (CMAP) was set at the beginning of the surgery and values of <50% CMAP alerted the surgeon to stop the manipulation until the amplitude normalized. Intraoperative nerve injury was correctly recognized in all three cases and a direct end-to-end neurorrhaphy was performed, enabling a long-term recovery that reached HB grade I/II in these patients.

### acIONM in transcranial stimulation during posterior fossa surgery

Multipulse transcranial electric stimulation (TES) of the corticobulbar pathway during posterior cranial fossa surgery is a method of continuous monitoring of the functioning of the facial nerve through analysis of the muscle motor evoked potential (FNMEP). The stimulator, in the form of a cup electrode, is placed above the skull. This method uses clusters of three to four pulses of current, producing a supramaximal stimulation (100–400 V) with an interpulse interval of 1–2 ms and a cluster frequency of 5.6–3.3 × 10^−3^ Hz [6]. In a study by Dong et al., no patient with a final amplitude of 50% or greater than the baseline amplitude had more than a mild deterioration in facial nerve function when compared with their preoperative facial nerve function [6].

### acIONM of the vestibulocochlear nerve during posterior fossa surgery

Neuromonitoring of the vestibulocochlear nerve using brain stem auditory evoked potentials (BAEP) during cerebellopontine angle surgery can also be described as acIONM. During the preparation, acoustic clicks of 100–110 dB are continuously delivered to the ear in the proximity of the eighth cranial nerve by means of ear pods. JEWETT waves I and V deliver the most useful information owing to their constancy. To some extent, wave III can also be used for interpretation. The other ear receives white noise of 60–70 dB in order to mask the contralateral clicks. A similar method used in the same type of surgery is electrocochleography (ECochG) acIONM, which obtains a waveform equivalent to JEWETT wave I of the BAEP, however with significantly higher amplitude. Here, compound action potential (CAP) should also be noted. This method takes advantage of an electrode placed either between the tumor and the root entry of the nerve into the brain stem [41] or distal to the tumor [11]. The BAEP, ECochG, and CAP are complementary methods that do not exclude one another but, on the contrary, are commonly used simultaneously.

The BAEP acIONM has shown reliable results in predicting postoperative hearing function. For example, Neu [19] stratified patients monitored by acIONM using BAEP into four groups. All patients with stable wave V (pattern 1) showed definite hearing preservation while all patients with irreversible abrupt loss of BAEP (pattern 2) lost their hearing, despite early hearing preservation in two cases. All patients with irreversible progressive loss of either wave I or wave V (pattern 3) eventually suffered from definite postoperative hearing loss, despite early hearing preservation in two cases. Those cases with intraoperative reversible loss of BAEP (pattern 4) showed variable short- and long-term hearing outcomes [19]. In a study by Yamakami, BAEP and CAP were used concomitantly [41]. Reliable BAEP values referring to wave V could be obtained only in 41% of patients, whereas reproducible CAP without artifacts were noted in 91% of patients. All patients who preserved CAP on completion of a microsurgical tumor removal preserved serviceable hearing postoperatively, showing a 100% specificity and sensitivity [41].

### pcIONM of the facial nerve during posterior fossa surgery

In contrast to acIONM, methods of continuous monitoring that can be described as passive cIONM have evolved. These rely purely on analyzing the discharge patterns that occur during the operation. Such “free-running EMG” is used in neurosurgery in the monitoring of the facial nerve. In 1986, Prass and Lüders described spikes, bursts, and three types of trains in the EMG signal during posterior fossa surgery on 30 patients [22]. Trains represented sustained periodic EMG activity that lasted for seconds. The presence of A‑trains [22], roughly referring to high-frequency and low-amplitude sinusoidal EMG patterns, were later correlated with a lower postoperative HB score [26]. Prell processed the train time in the signal obtained on a computer in an automated fashion offline [24]. This evolved into software that was used in an online fashion in the operating theater [23]. The software enabled a real-time quantification of train time, informing the surgeon on the “cumulative damage” to the nerve [23]. By having real-time information on the impending nerve injury, the surgeon could estimate the probable postoperative nerve function and actively change the operative strategy in order to avoid further deterioration of the nerve function.

Signal recovery of ≥50% after LOS correlates with normal postoperative nerve function

The information the surgeon obtained was represented in analogy to a traffic light. The status of the nerve remained in the “green area” when the train time remained under 0.125 s, which meant the dissection could be continued safely. When the train time exceeded 0.125 s but remained under 2.5 s the light was changed to “orange,” which indicated the need for increased care because this amount of train time accounted for a deterioration in the HB scale to the third grade in 25% of patients with normal preoperative facial nerve function. An excess of train time beyond 2.5 s resulted in the light changing to “red” and was clearly associated with a significant increase in paresis, which prompted the surgeon to abort the manipulation and re-evaluate the surgical plan. For example, the angle and site of further preparation was changed, nimodipine was applied intraoperatively, resection was stopped in selected patients, and a revision procedure was scheduled.

## Safety of cIONM

The safety of cIONM is of paramount importance. Passive cIONM can be excluded from precaution for obvious reasons: This method relies on the pure analysis of the signal that is noted and no active stimulation takes place, thus no nerve damage could possibly be done. This is not the case with acIONM where active stimulation is applied. The fact that one involves a novel method using active stimulation to the nerve makes the issue of safety of outmost importance.

Active cIONM has generally been described as a safe procedure in thyroid surgery throughout high-volume studies [8, 32, 33]. The safety of acIONM was demonstrated in a prospective study in which no heart rate variability and immunomodulatory effects were noted in spite of continuous stimulation of the vagus [8]. This was also observed in an earlier study by the same group of authors, where once again the distinct influence of acIONM on the autonomous nervous system balance was applied without alterations to heart rate, rhythm, or hemodynamic parameters [39]. In a study involving 102 patients, Phelan noted neither cases of adverse amplitude or latency changes, nor cases of adverse gastric, cardiac, pulmonary, or gastrointestinal side effects [21]. Active cIONM has been carried out safely also in patients with advanced atrioventricular block [28].

In an experimental study of 13 pigs by Lee, acIONM was applied using automated period stimulation in order to examine the force needed for a traction injury of the recurrent laryngeal nerve [15]. The nerves were deliberately stretched until loss of signal occurred. Seven days after the experiment, all nerves displayed EMG signal recovery, thus showing that acIONM alone does not induce structural damage to the nerve [15] and that recovery will take place.

However, some adverse effects have been reported [3, 18, 36]. In one patient, reversible vagal neuropraxia with visible perineural ecchymosis was caused by the APS® stimulating electrode (Automatic Periodic Stimulation, Medtronic, Jacksonville, FL, USA), as well as a subsequent inability to stimulate the nerve after the event. A short-term postoperative palsy was noted with reversal after 1 month. In the same publication, an allegedly serious hemodynamic instability (bradycardia and hypotension) was noted after the onset of acIONM in a young healthy patient with no history of cardiac events. The effect could be promptly reversed by removing the electrode. Upon replacement of the electrode, the exact same effect was again noted, and again was rapidly reversed by removing the electrode once more. Patient recruitment in the study was abandoned after these two adverse events [36].

Two studies together comprising approximately 250 nerves at risk reported an identical risk of 2% for vagus nerve injury due to the placement of the APS® electrode [3, 18]. The events were not associated with permanent postoperative nerve palsy.

### Safe application of acIONM in children

There are also studies regarding the safety of acIONM in children. For example, Bozinov used transcranial electric stimulation to continuously monitor FNMEP in 21 patients (median age: 5.5 years; range: 5 months to 15 years). The FNMEP was also feasible and safe in the young population, with similar values achieved in the prediction of postoperative facial nerve function to those of adults reported in other studies. The HB grade remained the same pre- and postoperatively in 23 of 24 surgeries. The presence of FNMEP influenced the surgical strategy and contributed to tumor resection in those cases where direct nerve stimulation gave no muscle response [2]. A second example of acIONM in children is the previously mentioned study by Ulkatan where percutaneous stimulation was applied in 201 surgeries on 161 patients aged 14 ± 14 years [38]. Safe application of acIONM in thyroid surgery was demonstrated in a large-scale study including 105 children [29].

## Limitations of cIONM

Aside from the aforementioned rare adverse effects reported due to electrode placement, some other limitations come to mind. In reference to our own experience with neuromonitoring, one must note the problem of technical artifacts and false-positive or false-negative alarms that often occur. The field of cIONM, with real-time nerve surveillance, emphasizes the burden of such false alarms. All the aforementioned studies share the same problem of a low positive predictive value. In this context, for example, in thyroid surgery, a false-positive alarm can be responsible for unnecessarily delaying the procedure of the contralateral side.

To date, no studies have applied cIONM in parotid surgery

Apart from rare retrospective studies comparing cIONM with iIONM in thyroid surgery, there are no studies comparing this novel method with the conventional iIONM in the other fields of application mentioned earlier. Prospective randomized studies are urgently needed in order to prove the claimed benefits.

Some of the methods described remain “single-study reports,” meaning that the method failed to become establish in clinical practice(see Sects. “Direct acIONM in posterior cranial fossa surgery” and “acIONM in vascular malformation surgery”). In some studies, only the last acquired amplitude and the baseline amplitude were used for statistical evaluation (see Sect. “Direct acIONM in posterior cranial fossa surgery”), in others the frequency of continuous stimulation was only one in 3–5 min (see Sect. “acIONM in transcranial stimulation during posterior fossa surgery”), questioning the need for continuous stimulation.

## Practical conclusion

- The novel methods of continuous intraoperative neuromonitoring (cIONM) have made the recognition of impending nerve injury and the consequent change of operative strategy possible.
- Furthermore, the prediction of postoperative nerve function based on cIONM has been refined in comparison to that of intermittent intraoperative neuromonitoring (iIONM).
- Continuous IONM brings a new dimension to the field of neuromonitoring.
- It is a new evolving instrument destined to help surgeons in performing surgical maneuvers in close proximity to neural structures.
- It cannot and does not replace good operative technique and patency; however, it does provide reliable and safe assistance.
- The safety of active cIONM (acIONM) has been demonstrated in animal and human studies.
- The field appears to be open for future studies, especially in surgery of the parotid gland where to date neither acIONM nor pcIONM have been applied.
